# Monoaminergic Mechanisms in Epilepsy May Offer Innovative Therapeutic Opportunity for Monoaminergic Multi-Target Drugs

**DOI:** 10.3389/fnins.2016.00492

**Published:** 2016-11-10

**Authors:** Dubravka Svob Strac, Nela Pivac, Ilse J. Smolders, Wieslawa A. Fogel, Philippe De Deurwaerdere, Giuseppe Di Giovanni

**Affiliations:** ^1^Division of Molecular Medicine, Rudjer Boskovic InstituteZagreb, Croatia; ^2^Department of Pharmaceutical Chemistry and Drug Analysis, Vrije Universiteit BrusselBrussels, Belgium; ^3^Department of Hormone Biochemistry, Medical University of LodzLodz, Poland; ^4^Centre National de la Recherche Scientifique (Unité Mixte de Recherche 5293)Bordeaux, France; ^5^Laboratory of Neurophysiology, Department of Physiology and Biochemistry, University of MaltaMsida, Malta

**Keywords:** monoamine receptors, multi-target direct ligands, epilepsy, epileptogenesis, antiepileptic drugs, quad-partite synapse, astrocytes, microglia

## Abstract

A large body of experimental and clinical evidence has strongly suggested that monoamines play an important role in regulating epileptogenesis, seizure susceptibility, convulsions, and comorbid psychiatric disorders commonly seen in people with epilepsy (PWE). However, neither the relative significance of individual monoamines nor their interaction has yet been fully clarified due to the complexity of these neurotransmitter systems. In addition, epilepsy is diverse, with many different seizure types and epilepsy syndromes, and the role played by monoamines may vary from one condition to another. In this review, we will focus on the role of serotonin, dopamine, noradrenaline, histamine, and melatonin in epilepsy. Recent experimental, clinical, and genetic evidence will be reviewed in consideration of the mutual relationship of monoamines with the other putative neurotransmitters. The complexity of epileptic pathogenesis may explain why the currently available drugs, developed according to the classic drug discovery paradigm of “one-molecule-one-target,” have turned out to be effective only in a percentage of PWE. Although, no antiepileptic drugs currently target specifically monoaminergic systems, multi-target directed ligands acting on different monoaminergic proteins, present on both neurons and glia cells, may represent a new approach in the management of seizures, and their generation as well as comorbid neuropsychiatric disorders.

## Introduction

Epilepsy is a complex chronic group of neurological disorders that affects ~60 million people worldwide, with 6 million in Europe alone (Baulac et al., 2015).

Epilepsy is characterized by spontaneous and recurrent unprovoked seizures (bursts of neuronal hyperactivity) arising in the brain that can be “focal” or “partial” if they remain confined to their area of origin, or “generalized” if they spread to the entire cerebral hemispheres. Recently, seizures have been classified in focal and generalized convulsive and non-convulsive epilepsies according to their different electrophysiological and clinical characteristics (Berg et al., 2010). Epilepsy can be symptomatic, for example, due to stroke, infections, brain tumors, prolonged febrile seizures, and other occurrences of status epilepticus (SE). Additionally, about 40% of all epilepsies, especially during childhood, and adolescence (Guerrini, 2006), are idiopathic epilepsies. Several defects in ion channel or neurotransmitter genes or proteins that control brain excitability have been recently identified in some idiopathic epilepsies (Scharfman, 2007). In addition, various epidemiological and family studies have suggested a genetic basis of epilepsy (Myers and Mefford, 2015). A number of genes have been associated with epilepsy disorders in a Mendelian manner (Harden, 2002). However, it has been suggested that most epilepsies have a polygenic basis, with multiple genetic susceptibility factors which have only partial effects, but act in concert, and interact with various environmental factors (Ferraro and Buono, 2006; Tan and Berkovic, 2010). The genes associated with epilepsy are involved in different molecular pathways, including the regulation of development and function of the nervous system (Holmes and Noebels, 2016). Although, the majority of genes associated with epilepsies are coding for different voltage and ligand-gated ion channels or regulating the action of excitatory or inhibitory neurotransmission (i.e., CHRNA4, CHRNA2, CHRNB2, GABRG2, GABRA1, KCNQ2, KCNQ3, SCN1B, SCN1A, SCN2A), the potential role of several other genes (i.e., ARX, CDKL5, LGI1, PCDH19, SLC2A1, SPTAN1, STXBP1) in the epilepsy has also been also suggested (Rees, 2010; Hildebrand et al., 2013).

Genetics therefore plays a role, although a complex one, in almost all acquired epilepsies.

The lifetime prevalence of epilepsy is 1–2%, and it affects individuals of all ages regardless of gender or socio-economic status. Epilepsy is a significant health concern for the human population and people with epilepsy (PWE) carry a risk of premature mortality, with a life expectancy 10 years less than the general population (Gaitatzis et al., 2004).

There is currently no cure or prevention for epilepsy. Most, if not all of the approved antiepileptic drugs (AEDs) are not truly “antiepileptic” but merely “anti-seizures” (Van Liefferinge et al., 2013). Indeed, the AEDs do not stop epileptogenesis, the process of converting a normal brain to a brain with epilepsy, but at the most they reach complete seizure control. Unfortunately, not all PWE respond to the therapies, with 30–40% of them possessing pharmacoresistant epilepsy (Kobau et al., 2008). Although, the efforts in antiepileptic drug development have not solved the issue, they have encouraged experimental and clinical research to focus on different mechanisms involved in the neurological disorder. Indeed, many candidate processes and molecular targets are currently under intense scrutiny and hopefully will improve treatment and quality of life of PWE.

Monoamines are major neuromodulator systems in the central nervous system (CNS) and compelling evidence accumulated in the last 30 years has also established their pivotal role in epilepsy (Kobayashi and Mori, 1977; Kurian et al., 2011). Serotonin (5-HT; Bagdy et al., 2007; Guiard and Di Giovanni, 2015), dopamine (DA; Bozzi and Borrelli, 2013), noradrenaline (NA; Giorgi et al., 2004), histamine (Bhowmik et al., 2012), and melatonin (MT; Tchekalarova et al., 2015b; Brigo and Igwe, 2016) are all known to halt seizure activity.

Further proof of monoaminergic involvement in the pathogenesis of epilepsy is the evidence that depression, bipolar disorders, and other neuropsychiatric disorders classically related to monoamine dysfunctions, may augment the risk of seizures and/or vice versa. As matter of fact, PWE with longer duration of active epilepsy show higher comorbidity of depressive disorders, bipolar disorder and anxiety (Rocha et al., 2014), and in depressed patients there is a higher rate of epilepsy compared to general population (Garcia, 2012). It has been suggested that epilepsy and mood disorders may be different manifestations of the same disturbances in transmission and/or signal transduction mediated by monoamines, hyperactivity of the hypothalamic-pituitary-adrenal axis, and CNS inflammation (Rocha et al., 2014). As both epilepsy and monoamine-based neuropsychiatric disorders are complex diseases that imply changes in multiple neurotransmitters and both neuronal and glial cells activity, a comprehensive understanding of the underlying mechanisms is still in its infancy. Nevertheless, this evidence of dual link between these two disorders suggest that drugs targeting monoamines may be useful for both epilepsy and its neuropsychiatric comorbidities (Guiard and Di Giovanni, 2015; Venzi et al., 2016).

Although, the role of monoamines in epilepsy was reviewed for the first time by Kobayashi and Mori (1977), followed by intensive exploration in pre-clinical and clinical research over the last 40 years, this has not led to new treatments. Indeed, the questions asked by Kobayashi and Mori (1977) “Is there an abnormal metabolism of monoamines in the brain of epileptic patients? If so, how is it related to the elaboration or maintenance of epileptic seizures?” do not yet have definitive answers.

Compelling evidence shows that monoaminergic systems appear dysregulated in animal (Szabo et al., 2015) and human epileptic brain and increased monoamines and metabolite levels in the cerebrospinal fluid (CSF) of PWE have been consistently observed (Pintor et al., 1990; Naffah-Mazzacoratti et al., 1996).

Nevertheless, the elevated levels of 5-HT and DA metabolites during epilepsy may represent an epiphenomenon, rather than a concerted strategy of local or distal neurons to contain an epileptogenic focus (Lowy and Meltzer, 1988). Indeed, the rate of monoaminergic metabolism (i.e., synthesis, uptake, and clearance) does not significantly correlate with the epileptic condition in baboon (Szabo et al., 2015). Moreover, it has recently been shown that receptor antagonism completely prevented all kainic acid-induced increases in extracellular hippocampal 5-HT levels in rats without affecting seizure development *per se*. This result suggested a lack of a direct relationship between seizure susceptibility and alterations in hippocampal 5-HT levels, at least in this rat model (Tchekalarova et al., 2015b).

These findings, however, do not necessarily exclude the monoaminergic system as a potential source of pathogenesis in epilepsy and sudden unexpected death in epilepsy (SUDEP; Richerson and Buchanan, 2011).

As a further complication, monoamines seem to have a dual effect being proconvulsant when in high concentration in the epileptic foci. Indeed, within a certain concentration range, intrahippocampally applied 5-HT contributed to the prevention of hippocampally evoked limbic seizures. On the other hand, excessive 5-HT increases worsened seizure outcome (Clinckers et al., 2004) and elevated, endogenous noradrenergic transmission is for example an etiological factor in some cases of epilepsy (Fitzgerald, 2010).

In the following sections of this review, we will focus on the role of different monoamines in seizure onset and spread, discussing anatomical, pharmacological, and genetic evidence obtained in animal and human studies. We will provide the rationale for the use of drugs targeting monoamines or their related molecules in epilepsy, some already representing good examples of multi-target directed drugs. We finish by exploring the interesting possibility that the monoaminergic treatment may cure the dysfunction of the quad-partite synapse acting at the level of their different components, i.e., (pre- and postsynaptic) neurons, astrocytes, and microglia cells.

## Monoamines in epilepsy: preclinical and clinical evidence

### Serotonergic system in epilepsy

It goes without saying that 5-HT is involved in epilepsy mechanisms. According to a variety of recent findings, neurodevelopmental alterations of serotonergic circuits in mice are crucial in controlling seizure susceptibility to the well-established chemoconvulsant kainic acid (KA; i.e., a glutamatergic kainate receptor agonist) in later life (Tripathi and Bozzi, 2015). Clinical presentations of human epilepsy have often been attributed to deficiencies of cerebral monoamines, including 5-HT (Kurian et al., 2011). Serotonin (but also DA) enhancement may even be involved to a certain extent in the mechanisms of action of several clinically used antiepileptic drugs (Yan et al., 1992; Ahmad et al., 2005; Biton, 2007).

Nevertheless, the picture is not always that clear-cut. The classical view is that the monoamine enhancing antidepressant drugs are contraindicated in PWE, or should at least be used with caution. Against this assumption, more and more reports provided evidence that several 5-HT enhancing antidepressants were not proconvulsant but rather displayed anticonvulsant properties (Hamid and Kanner, 2013). Dailey and Naritoku (1996) deducted that non-monoaminergic off-target effects of antidepressants are most likely responsible for the increased risk of seizures.

The most straightforward answer to the question—why 5-HT seems to exert such a complex role in the modulation of enhanced brain excitability and epilepsy phenomena—is of course the fact that 5-HT interacts with a variety of different receptor subtypes linked to divergent signal transduction cascades, thereby often exerting opposing control on cell membrane potentials (De Deurwaerdere and Di Giovanni, 2016). Moreover, these 5-HTR subtypes are differently distributed in distinct brain areas and diverse brain circuitries involved in various types of epilepsy. Moreover, as will be illustrated in following sections, the same holds true for the other monoamines described within this review.

For the remainder of this 5-HT section, we will focus merely on 5-HTR subtype-specific seizure-modulating actions. Most evidence can be found on the roles of 5-HT_1_R and 5-HT_2_R subtypes and the 5-HT_3_R, while—to the best of our knowledge—less literature is available with regard to the possible involvement of 5-HT_4_ and 5-HT_6_Rs in epilepsy mechanisms. No data has been published on 5-HT_5_Rs in epilepsy. Finally, the involvement of the 5-HT_7_R in mechanisms of epilepsy is still ambiguous (Ciranna and Catania, 2014; Nikiforuk, 2015).

Within the scope of the current manuscript, it will be impossible to review all the available data to date, but we will focus on the most prominent and/or recent findings. We also refer to the review paper by Panczyk et al. (2015) who listed the evidence for the involvement of 5-HT_1A_, 5-HT_2C_, 5-HT_3_, 5-HT_4_, and 5-HT_7_Rs as well as the 5-HT transporter (SERT) in epilepsy (Panczyk et al., 2015).

With regard to the 5-HT_2A_R and its seizure modulating effects, literature is abundant but also very complex. For a complete and recent overview on the role of the 5-HT_2A_R in rodent epilepsy models, we refer to the detailed review by Guiard and Di Giovanni (2015). They summarized the evidence for 5-HT_2A_R modulation in both generalized and focal epilepsies, and concluded that both proconvulsant and anticonvulsant roles have been established for this 5-HT_2A_R subtype, depending on the dose of the ligands used, the experimental rodent model investigated and the different populations of the receptors. At high doses of the 5-HT_2A_R ligands, proconvulsant effects were often noted which may be attributed—at least partly—to other non-selective off-target effects (Guiard and Di Giovanni, 2015). Because of this complexity, we refer the readers to this in deep review.

#### Focal seizures

##### Human data

In PWE suffering from TLE, hippocampal 5-HT depletion (da Fonseca et al., 2015) and reduced 5-HT_1A_R availability have been observed. The latter somatodendritic 5-HT_1A_ autoreceptor is one of the best characterized subtypes of the 14 known 5-HTRs and is clearly implicated in seizure modulation. A large body of evidence on this receptor subtype in epilepsy has arisen from many positron emission tomography (PET) studies in PWE, and reduced 5-HT_1A_R binding in the epileptic focus has been repeatedly and consistently found in these temporal lobe epilepsy (TLE) patients (Hasler et al., 2007; Lothe et al., 2008; Giovacchini et al., 2009; Assem-Hilger et al., 2010). All of these studies point to the fact that diminished 5-HT_1A_R expression and subsequent less activation by endogenous 5-HT may lead to the epileptic phenotype. PET imaging of brain 5-HT_1*A*_Rs has also helped in the correct identification of the epileptogenic zone during the preoperative evaluation of temporal lobe of PWE subjected to epilepsy surgery (Didelot et al., 2008; Theodore et al., 2012).

More recently, it has been shown that both 5-HT_6_Rs (Wang et al., 2015) and 5-HT_7_Rs (Yang et al., 2012) were upregulated in the human neocortex of PWE with refractory TLE. These interesting findings call for more studies with 5-HT_6_R and 5-HT_7_R ligands.

##### Animal data

Acute seizure evocation with KA led to increases in hippocampal 5-HT tissue content and extracellular 5-HT levels (Alfaro-Rodriguez et al., 2011; Tchekalarova et al., 2015a) while during the spontaneous recurrent limbic seizures in the KA model decreases in 5-HT content were found (Tchekalarova et al., 2011). In another well-established post-SE rat model for focal epilepsy using pilocarpine (i.e., a muscarinergic receptor agonist) as the chemoconvulsant, 5-HT hippocampal content (Cavalheiro et al., 1994) and hippocampal 5-HT levels (Meurs et al., 2008) were increased during the acute seizure phase but not during the following spontaneous recurrent seizure phase (Cavalheiro et al., 1994; Szyndler et al., 2005). Comparing three acute limbic seizure models, which differed only in the chemoconvulsant used to evoke the seizures in rats, no straightforward correlation between the seizure activity and increased hippocampal extracellular 5-HT concentrations could be found (Meurs et al., 2008).

Concerning the role of the somatodendritic 5-HT_1A_ autoreceptor in focal epilepsy, the majority of pharmacological studies clearly highlight the anticonvulsant effects of 5-HT_1A_R agonists against limbic seizures evoked in various rat models, e.g., against pilocarpine-induced seizures (Clinckers et al., 2004; Lopez-Meraz et al., 2005; Pericic et al., 2005; Orban et al., 2013), as well as against status epilepticus evoked by lithium pilocarpine (Yang et al., 2014).

Activation of the 5-HT_2C_Rs do not appear to play a pivotal role in focal epilepsy or on the contrary is proepileptic (Di Giovanni and De Deurwaerdere, 2016). Indeed, 5-HT_2C_R agonists with different pharmacological profiles such as meta-chlorophenylpiperazine (mCPP) and lorcaserin, but not RO60-0175, were able to stop the elongation of the electrically triggered hippocampal maximal dentate gyrus activation in a limbic seizure model in anesthetized rats, an effect that was not blocked but rather potentiated by pre-treatment of SB 242084 (Orban et al., 2014), a selective 5-HT_2C_R antagonist. In addition, 5-HT_3_Rs display also no importance in focal hippocampal seizures (Watanabe et al., 1998).

A selective 5-HT_6_R antagonist was able to attenuate spontaneous recurrent seizures in the post-SE pilocarpine rat model, and diminished hippocampal mechanistic target of rapamycin (mTOR) activity, suggesting that 5-HT_6_Rs may mediate limbic seizures via mTOR signaling (Wang et al., 2015). Moreover, 5-HT_6_R expression was upregulated in the hippocampus and neocortex of the pilocarpine-treated rats (Wang et al., 2015), confirming the finding in PWE as described above. There is one study showing that 5-HT_7_R antagonism also diminished the number of limbic seizures in pilocarpine-treated rats (Yang et al., 2012). More confirmatory results with 5-HT_6_R and 5-HT_7_R antagonists in focal epilepsy models might be interesting to obtain.

##### *In vitro* data

Serotonin inhibited bicuculline (i.e., a GABA_*A*_ receptor antagonist)- and KA-evoked epileptiform activity in brain slices via membrane hyperpolarization (Salgado and Alkadhi, 1995). The use of a 5-HT_3_R agonist showed no effect on cortical epileptiform activity (Bobula et al., 2001). In rat hippocampal brain slices, 5-HT_4_R agonism aggravated population spikes, evoked by electrical stimulation and spontaneous epileptiform activity (Tokarski et al., 2002). The influence of many other 5-HT receptor subtypes on epileptiform activity remains elusive.

### Generalized convulsive seizures

#### Animal data

Hippocampal 5-HT_1A_ and 5-HT_1B_R immunoreactivities were decreased in the rat unilateral hypoxic-induced epilepsy model (An and Kim, 2011). Anticonvulsant effects of 5-HT_1A_R agonists have also been repeatedly reported in models for generalized seizures, such as the pentylenetretrazole (PTZ, a prototypic antagonist of GABA_A_ receptors) model (Lopez-Meraz et al., 2005), tonic-clonic seizures evoked by amygdala kindling (Lopez-Meraz et al., 2005), and the picrotoxin (another typically used antagonist of GABA_A_ receptors) model (Peričić et al., 2005). The seizure modulating roles of specific 5-HT_1B_, 5-HT_1D_, and 5-HT_1E_Rs are less studied, but anticonvulsant properties upon 5-HT_1B_ activation in the PTZ model were described (Wesolowska et al., 2006).

Strong evidence for decreased excitability upon 5-HT_2*C*_R activation was obtained from the 5-HT_2C_R knock out (KO) mice that displayed a clear generalized epileptic phenotype and exhibited an increased sensitivity to chemoconvulsant PTZ (Tecott et al., 1995; Heisler et al., 1998).

The first report using a 5-HT_3_R ligand in relation to epilepsy was described by Cutler and Piper (Cutler, 1990) who showed that 5-HT_3_R antagonism had no effects upon seizure susceptibility or severity in Mongolian gerbils. Unclear effects of 5-HT_3_R antagonists were noted on audiogenic seizures in Dilute Brown Non-Agouti (DBA)/2 mice (Semenova and Ticku, 1992) and on alcohol withdrawal seizures (Kostowski et al., 1993; Grant et al., 1994). A 5-HT_3_R agonist facilitated generalized seizure development in the well-characterized rat amygdala kindling model (Wada et al., 1997). Despite all these initial negative results, recent interest in the 5-HT_3_R subtype emerged in the PTZ model for generalized seizures and in PTZ kindling. Indeed, 5-HT_3_R agonism exhibited dose-dependent anticonvulsant effects in the PTZ model (Li et al., 2014). Moreover, the 5-HT_3_R subtype seems to play a prominent role in mediating the anticonvulsant effects of various selective 5-HT reuptake inhibitors in this classical PTZ model for generalized epilepsy (Payandemehr et al., 2012; Alhaj et al., 2015).

PTZ-induced convulsive responses were aggravated in 5-HT_4_R KO mice (Compan et al., 2004). Potent and selective 5-HT_6_R antagonists displayed clear anticonvulsant effects in the maximal electroshock test in rats (Routledge et al., 2000; Stean et al., 2002; Hirst et al., 2006).

Some pharmacological studies with 5-HT_7_R antagonists pointed to anticonvulsant effects in various rodent models. For instance, antagonism of 5-HT_7_Rs protected DBA/2J mice against audiogenic seizures (Bourson et al., 1997). Anticonvulsant effects of 5-HT_7_R agonists were also described against picrotoxin-evoked seizures in mice (Pericic and Svob Strac, 2007). In line with these findings, constitutive deletion of the 5-HT_7_R resulted in proconvulsant effects as the KO mice exhibited decreased thresholds for electroshock-induced seizures and decreased seizure thresholds for PTZ- and cocaine-induced seizures (Witkin et al., 2007). More investigations are therefore needed to clarify the exact role of the 5-HT_7_R in generalized epilepsy.

### Generalized non-convulsive seizures and epilepsy syndromes

#### Human data

Insufficient human evidence on generalized non-convulsive seizures and epilepsy syndromes exists so far. Treatment of a male patient suffering from drug-resistant epilepsy, resulting from a deleterious *de novo* sodium voltage-gated channel alpha subunit 2 (SCN2A), gene splice-site mutation, with the 5-HT precursor 5-hydroxytryptophan, led to mild clinical improvement (Horvath et al., 2016).

#### Animal data

Some typical 5-HT_2C_R agonists dose-dependently suppressed absence seizures in the Genetic Absence Epilepsy Rats from Strasbourg (GAERS), a well-established polygenic rat model of absence epilepsy and non-convulsive seizures; these effects were prevented when administering a selective 5-HT_2*C*_R antagonist, indicating the potential of selective 5-HT_2C_R agonists as novel anti-absence drugs (Venzi et al., 2016). Experiments on Wistar Albino Glaxo rats from Rijswijk (WAG/Rij) rats, another polygenic rat model of absence epilepsy, have found that mCPP decreased spike-wave discharges (SWDs) cumulative duration via the activation of 5-HT_2C_Rs (Jakus et al., 2003). Strikingly, while SB 242084 had no effect on SWDs when administered on its own in WAG/Rij rats, (Jakus et al., 2003; Jakus and Bagdy, 2011) it showed some anti-absence effects in GAERS. The 5-HT_2B_R is less characterized and/or without effect on the threshold for generalized seizures (Upton et al., 1998; Di Giovanni and De Deurwaerdere, 2016). Antagonism of 5-HT_7_Rs reduced spontaneous spike-wave discharges in the WAG/Rij rats (Graf et al., 2004).

#### *In vitro* data

Sourbron et al. (2016) were able to demonstrate that selective 5-HT_1D_-, 5-HT_1E_-, 5-HT_2A_-, 5-HT_2C_-, and 5-HT_7_-R agonists significantly decreased epileptiform activity in a homozygous sodium voltage gated channel alpha subunit 1 (SCN1A) mutant zebrafish model for Dravet syndrome (Sourbron et al., 2016).

### Dopaminergic system in epilepsy

The seizure modulating effects of DA have received a lot of attention since the 1960s, so it is almost an impossible task to review the abundant evidence to date. This section will therefore summarize the most obvious findings and highlight a few recent studies. For a more expanded review, we recommend the fine manuscript by Bozzi and Borrelli (2013) who reviewed the intracellular signaling pathways triggered by activation of different DA receptors (DARs) in relation to their role in limbic seizures and epileptogenesis (Bozzi and Borrelli, 2013).

For years, it has been known that innate deficiencies in DA contributed to the seizure-prone states of some genetic rodent models and therefore may be a predisposing factor for human epilepsy (Starr, 1996). Generally, excitability is affected in a biphasic fashion via DAergic actions: D_1_-like receptor activation merely increases excitation while D_2_-like receptor activation largely leads to anticonvulsant actions. The important role of D_2_-like receptors in regulating brain excitability is clinically supported by the well-known decrease in the seizure thresholds in PWE treated with antipsychotic D_2_R antagonists. However, information on selective D_3_R, D_4_R, and D_5_R modulating effects on seizures are scarce.

#### Focal seizures

##### Human data

In PWE suffering from TLE, alterations in the neocortical DA content, D1-like and D2-like receptor expression, and DA transporter (DAT) binding have been reported (Rocha et al., 2012). Reduced D_2_R/D_3_R binding, sustained impairment of the DAergic system, was demonstrated in extrastriatal and/or striatal brain regions of PWE, specifically with TLE (Bernedo Paredes et al., 2015).

##### Animal data

Similarly as described above for 5-HT, acute kainic acid-induced seizures increased hippocampal DA tissue content and extracellular DA levels, (Alfaro-Rodriguez et al., 2011; Tchekalarova et al., 2015b) while during the spontaneous recurrent limbic seizures in the kainic acid model, decreases in DA content were found (Tchekalarova et al., 2011). In the pilocarpine rat model, DA hippocampal content (Cavalheiro et al., 1994) and hippocampal DA levels (Meurs et al., 2008) were increased during the acute seizure phase. During the chronic recurrent seizure phase in the pilocarpine model, hippocampal DA content was elevated in one study (Cavalheiro et al., 1994) while no alterations were described in another study (Szyndler et al., 2005). Interestingly, when comparing three acute limbic seizure models, a direct relationship between the seizure activity and increased hippocampal extracellular concentrations of DA were established (Meurs et al., 2008).

In line with the majority of data described for generalized seizures, D_1_-like receptor-activation results in seizure enhancement in the limbic pilocarpine model (Barone et al., 1990). Activation of hippocampal D_2_-like receptors, leading to inhibition of adenylyl cyclase (AC) via Gi coupling, consistently protected rodents against limbic motor seizures, supporting seizure facilitation via D_1_R-mediated increases in cyclic adenosine monophosphate (cAMP; Bozzi and Borrelli, 2013). Moreover, the D_2_-like receptors seem to play a pivotal role in the overall anticonvulsant effect of hippocampal DA since a selective D_2_R antagonist abolished DA-mediated anticonvulsant effects in the acute pilocarpine limbic seizure model (Clinckers et al., 2004). Most interestingly, D_2_-like receptor signaling in the hippocampus also leads to glycogen synthase kinase 3β inhibition and hippocampal cell survival following kainic acid administration (Dunleavy et al., 2013).

##### *In vitro* data

Electrophysiological data demonstrated that DA can affect hippocampal excitability in a biphasic fashion but the predominant DA action was an D_2_-like receptor-mediated inhibitory effect via hyperpolarization of the resting membrane potential and a long-lasting increase in after-hypolarization (Benardo and Prince, 1982). D_4_R KO mice displayed cortical hyperexcitability, as measured with electrophysiological current and voltage-clamp recordings, suggesting that D_4_R activation can negatively modulate glutamate (GLU) activity in the frontal cortex (Rubinstein et al., 2001). This is not unexpected from a receptor from the D_2_-like receptor family that mainly exhibits decreased excitation upon activation.

#### Generalized convulsive seizures

##### Human data

Patients with juvenile myoclonic epilepsy showed a reduction in D_2_R/D_3_R binding restricted to the bilateral posterior putamen, suggesting an alteration of the DAergic system within this region (Landvogt et al., 2010).

##### Animal data

Repeated D_1_-like receptor activation results in generalized seizures, disrupted hippocampal plasticity, and impaired long-term recognition memory (Gangarossa et al., 2014). Initially, the majority of results on D_1_-like receptor agonist effects on behavioral seizure thresholds clearly indicated proconvulsant effects (Starr, 1996). Nevertheless, recent reports showed that D_1_-like receptor agonists, linked to stimulation of adenylate cyclase (AC; but not phospholipase C, PLC), led to prominent behavioral seizures in rodents, whereas D_1_-like receptor agonists, linked to stimulation of phospholipase C (PLC, but not AC), did not (O'Sullivan et al., 2008). The D_5_R belongs to the D_1_-like receptor family and upon its activation mainly increased excitation is observed, although less prominent in comparison with D_1_R activation and subsequent increases in cAMP (O'Sullivan et al., 2008).

The role of the D_3_R in seizure modulation appears to be more complex. D_3_R KOs were less sensitive to picrotoxin-induced clonic seizures and mortality suggesting proconvulsant D_3_R-mediated signaling (Micale et al., 2009). On the other hand, D_3_R-mediated agonist actions protected against acute and cocaine-kindled seizures in mice and reduced lethal effects of acute cocaine toxicity (Witkin et al., 2008). Most probably, different D_3_R downstream signaling cascades in different implicated brain regions may explain the contrasting results (Bozzi and Borrelli, 2013); however more investigations are required.

#### Generalized non-convulsive seizures and epilepsy syndromes

##### Human data

The DA precursor L-3,4-dihydroxyphenylalanine (L-DOPA) improved the clinical outcome of a male patient suffering from intractable epileptic encephalopathy (Horvath et al., 2016), again sustaining an overall anticonvulsant DA-mediated action. Nevertheless, DA concentrations in media collected from neural cultures, derived from induced pluripotent stem cells from a patient with the Dravet syndrome, were higher than those from wild-type neural cultures (Maeda et al., 2016).

##### Animal data

Activation of both D_1_-like and D_2_-like receptors showed anti-absence effects (Deransart et al., 2000), probably by decreasing GABA_A_ receptor-mediated tonic inhibition (Yague et al., 2013) that is altered in animal models of absence seizures (Cope et al., 2009). Up-regulation of D_3_ (but not D_1_, D_2_, or D_5_) receptor mRNA seems part of the epileptic phenotype in absence-epilepsy prone rats (Deransart et al., 2001). The role of D_1_-like and D_2_-like receptors in non-convulsive epilepsy is thus less clear-cut in comparison with the data obtained in focal and generalized seizure and epilepsy models.

### Noradrenergic system in epilepsy

The suggestion that NA may act as an anticonvulsant was posed over 60 years ago (Chen et al., 1954). Subsequent experimental studies provided firm evidence that the noradrenergic system modifies seizure activity. Nowadays, vagus nerve stimulation (VNS) is an adjunctive treatment for resistant epilepsy and depression (Panebianco et al., 2016).

#### Focal seizures

##### Human data

In this context, the receptor-binding assays with prazosin as a ligand, performed on isolated cortical cell membranes from 10 PWE subjected to temporal lobectomy due to intractable partial epilepsy, showed a reduced density of α_1_ adrenoceptor (AR) in the sites of the epileptic foci with no change in affinity. It was concluded that the lower receptor density may result in noradrenergic hyposensitivity that could contribute to a localized diminution in inhibitory mechanisms in epileptic foci (Briere et al., 1986).

##### Animal data

Likewise, VNS is an effective adjunctive treatment for medically refractory epilepsy, and was found to produce its anticonvulsive effect by increasing NA levels in the hippocampus that is critically involved in the generation of limbic seizures (Raedt et al., 2011). The anticonvulsant action of VNS on pilocarpine-induced seizures in rats can be abolished by the blockade of hippocampal α_2_-AR, indicating a strong causal link between the seizure-suppressing effect of VNS and hippocampal noradrenergic signaling (Raedt et al., 2011). Interestingly, combined but not separate α_2_- and β_2_-AR stimulation inhibited limbic seizures induced by pilocarpine infusion in the hippocampus of rats. On the other hand, α_1A_-AR stimulation and α_1D_-AR antagonism alone also inhibited seizures associated with respectively significant hippocampal GABA increases and GLU decreases (Clinckers et al., 2010).

##### *In vitro* data

NA has demonstrated both proconvulsant and antiepileptic properties; however, the specific pharmacology of these actions has not been clearly established. For instance, under conditions of impaired GABAergic inhibition, the excitatory and inhibitory effects of NA on hippocampal CA3 epileptiform activity are mediated primarily via β- and α_2_-ARs respectively. Moreover, the NA antiepileptic effect in CA3 epileptiform *in vitro* is not dependent on the increase in GABAergic function (Jurgens et al., 2005) but is due to the activation of the α_2_-AR on presynaptic glutamatergic terminals of the recurrent axon collaterals of the CA3 pyramidal neurons (Jurgens et al., 2007). While the α_1_-AR antagonists prazosin and terazosin had no effect on hippocampal CA3 epileptiform activity in slice with GABA system blocked, (Jurgens et al., 2005) there is *in vitro* evidence showing that α_1_-AR subtype activation was able to release GABA and somatostatin at the single cell level. This suggests that α_1_-AR activation may also represent one mechanism by which NA exerts anti-epileptic effects within the hippocampus.

#### Generalized convulsive seizures

##### Human data

NA may have proconvulsant and anticonvulsant properties under certain conditions; activated noradrenergic transmission could be an etiological factor in some epilepsies. The available clinical data on the subject (NA boosting antidepressants, α_2_ AR agonists, pheophromocytoma, etc.) are discussed in detail by Fitzgerald (Fitzgerald, 2010). It has been shown that co-administration of β-ARs ligands with conventional AEDs, i.e., diazepam, phenobarbital, lamotrigine, valproate, enhance the anticonvulsive efficacy of the latter ones (De Sarro et al., 2002; Fischer, 2002; Luchowska et al., 2002).

##### Animal data

It has been shown that the animals treated with the monoaminergic toxin 6-hydroxydopamine (6-OHDA) or the noradrenergic toxin N-(2-chloroethyl)-N-ethyl-2-bromobenzylamine (DSP4), as well as DA beta-hydroxylase (DBH) KO mice that lack NA, expressed increased susceptibility to convulsing stimuli (Bortolotto and Cavalheiro, 1986; McIntyre and Edson, 1989) while, on the contrary, the stimulation of the locus coeruleus in the same animals consistently reduced it (Libet et al., 1977; Weiss et al., 1990). Further confirmation of inhibitory effects of NA on epileptogenesis was obtained by studying the genetic epilepsy-prone rat (GEPR) model (Yan et al., 1993, 1998). With regard to a question related to the receptors involved, the studies yielded conflicting results; the same ligands—agonists or antagonists, could have proconvulsant or anticonvulsant effects, depending on the animal species, the strain, the model of epilepsy employed (for refs see Fitzgerald, 2010) and also receptor location, as in case of α_2_-AR (Szot et al., 2004). Specifically, in flurothyl model of generalized convulsive seizures, it has been suggested that presynaptic α_2_-AR is responsible for the proconvulsant effect of α_2_-AR agonists, while the postsynaptic α_2_-AR is responsible for the anticonvulsant effect of α_2_-AR agonists (Szot et al., 2004). That α_2_-ARs mediate anticonvulsive effects is supported also by the observation that D79N mice which carry a point mutation in the locus of α_2_-AR develop amygdala kindling very easily (Janumpalli et al., 1998). Fewer studies concerned with α_1_-AR have been conducted. In DBH KO mice, pre-treatment with α_1_-AR agonist protected these mice against PTZ induced seizures while α_1_-AR antagonist exacerbated PTZ induced seizures in the control mice (Weinshenker et al., 2001b). As to β-ARs, the anticonvulsive activity of propranolol, a non-selective antagonist, has been demonstrated in a variety of animal models of generalized tonic-clonic seizures. Propranolol reduced seizures induced in mice by lidocaine, PTZ, strychnine, low frequency, and maximal electroshock (Saelens et al., 1977; Akkan et al., 1989; Fischer, 2002) as well as by sound in DBA/2 mice (Anlezark et al., 1979; De Sarro et al., 2002), and increased the threshold for lidocaine-induced convulsions in awake animals (Nakamura et al., 2008). Other beta blockers that showed some protective effects include metoprolol, for instance against audio seizures (De Sarro et al., 2002). On the other hand, the anticonvulsant effects of higher doses of clenbuterol against generalized tonic-clonic seizures has also been demonstrated in a couple of used tests (Fischer et al., 2001). Pre-treatment with α_1_-AR or β_2_-AR, but not α_2_-AR or β_1_-AR agonist significantly protected against PTZ-induced seizures in DBH^−/−^ mice. Therefore, activation of the α_1_-AR is primarily responsible for the anticonvulsant activity of endogenous NA in the murine PTZ model of epilepsy. Endogenous NA probably does not activate the β_2_-AR under these conditions, but exogenous activation of the β_2_-AR produces an anticonvulsant effect (Weinshenker et al., 2001a).

#### Generalized non-convulsive seizures and epilepsy syndromes

##### Animal data

Further confirmation of inhibitory effects of NA on epileptogenesis was obtained in GAERS (Micheletti et al., 1987). It has also been shown that various antiepileptic drugs, i.e., carbamazepine, have a modulatory, activating effect on NA system (Olpe and Jones, 1983; Post, 1988).

### Melatonin system in epilepsy

Melatonin (MT), N-acetyl-5-methoxytryptamine, is a major hormone of the pineal gland chiefly involved in circadian and seasonal rhythm regulations. Beyond that, it exerts a multitude of anti-excitatory and sedating effects that have been reviewed recently (Reiter et al., 2010; Hardeland et al., 2011). The majority of data indicates anticonvulsant properties of MT when applied at pharmacological doses in both pre- and clinical investigations.

#### Focal seizures

##### *In vitro* data

In an early electrophysiological study, epileptiform field potentials were elicited by omission of Mg^2+^ from the superfusate and recorded from layers II–V of human temporal neocortical slices cut from tissue resected for surgical treatment of epilepsy. The frequency of occurrence of epileptiform field potentials was halved with application of MT (Fauteck et al., 1995).

#### Generalized convulsive seizures

##### Human data

Clinical studies on a group of 54 children have demonstrated that during a convulsive crisis, the MT concentration in blood, as measured in patient's serum, significantly peaked but normalized within 1 h. The MT production stimulated by the convulsive crisis may be part of the response of the organism counteracting the seizures effects (Molina-Carballo et al., 2007). Nevertheless, in other studies no changes in serum or salivary MT concentrations were found after epileptic seizures (Rao et al., 1989; Motta et al., 2014). Clinical reports have shown the beneficial effect of MT treatment on seizure activity during the day and night (Goldberg and Spealman, 1983; Peled et al., 2001) in patients with intractable epilepsy. Systematic review of all so far published clinical data on MT in relation to epilepsy, including therapeutic use of MT, lead to the conclusion that there is no marked improvement or worsening of seizures with MT (Jain and Besag, 2013) or its use as add-on treatment (Brigo and Igwe, 2016). Only large randomized double blind placebo-controlled trials could give the final answer. In addition, it has been recently suggested that melatonergic drugs may be effective in treating comorbid depression in PWE (Tchekalarova et al., 2015b)

##### Animal data

The anticonvulsant activity exerted by MT seen in animal models of epilepsy (Golombek et al., 1992, 1996; Cardinali et al., 2008; Solmaz et al., 2009) has been suggested to be executed via increasing the activity of GABAergic system (Golombek et al., 1996). A selective MT_1_/MT_2_R agonist mimicked the MT effects in rat rapid kindling model and in the spontaneously epileptic mice lacking voltage-gated Kv1.1 channels (Kcna1-null mice; Fenoglio-Simeone et al., 2009). Beneficial actions of MT in epilepsy have also been attributed to its free radical scavenging properties (Mohanan and Yamamoto, 2002). MT has been reported to have an anticonvulsant action in many models of acute seizures, such as those produced by the administration of PTZ, picrotoxin, bicuculline, pilocarpine, l-cysteine, kainate, 3-mercaptopropionic acid, quinolinate, GLU, strychnine, N-methyl-d-aspartate (NMDA), or penicillin, as well as in the maximal electroshock seizure (MES) test in rats, mice, gerbils, and hamsters (see Banach et al., 2011). MT treatment during epileptogenesis can have beneficial effects against the deleterious consequences of SE in the KA model of TLE. Melatonin chronic treatment increased the seizure-latent period, decreased the frequency of spontaneous recurrent seizures (SRSs), and attenuated the circadian rhythm of seizure activity (Petkova et al., 2014; Tchekalarova et al., 2015b). These findings are in agreement with the earlier evidence that pinealectomy facilitates the epileptogenic process that follows the long-lasting SE. This facilitation can be partially reverted by the simultaneous administration of MT (De Lima et al., 2005). Agomelatine is a novel antidepressant agent, which is structurally homologous to MT. It is a potent MT1 and MT2 MTR agonist as well as a 5-HT_2C_R receptor antagonist (Millan et al., 2003). It was recently approved as an antidepressant medication with comparable efficacy to classical antidepressant drugs (Sansone and Sansone, 2011). Agomelatine has anticonvulsant activity shown in PTZ- or pilocarpine-induced seizure models due to its combined action at MT1/2 and 5-HT_2C_Rs (Aguiar et al., 2012).

#### Generalized non-convulsive seizures and epilepsy syndromes

##### Animal data

It has been shown that subchronic and systemic administration of agomelatine and MT displayed considerable antiepileptic effects on absence seizures in WAG/Rij rats (Hatice et al., 2015).

Agomelatine seems to be recommendable as a potential drug for absence epilepsy and many other complications such as depression and sleep disorders associated with epilepsy.

### Histaminergic system in epilepsy

The CNS histaminergic system is involved in variety of physiological and behavioral functions among them sleep-wake cycle, appetite control, cognitive functions, neuroendocrine functions, locomotor activity, emotion, and stress behavior. Histamine is formed locally in the CNS; the synthesizing enzyme histidine decarboxylase operates under subsaturating concentration of L-histidine. An increase in the substrate supply results in enhancement of cerebral histamine pool. Of the four histamine receptors (HRs), in the cerebral tissues H1–H_3_Rs are undoubtedly present. While H_1_ and H_2_Rs are located postsynaptically, the H_3_ ones are presynaptic auto- or heteroreceptors, controlling the release and synthesis of histamine, and modulating release of other neurotransmitters, e.g., acetylcholine, DA, NA, 5-HT, glutamate, and GABA (Schwartz et al., 1991; Haas and Panula, 2003). The involvement of cerebral histamine in regulation of seizure susceptibility is sufficiently documented by both clinical and experimental studies, which strongly point to histamine as an anticonvulsant. The antiepileptic activity seems to be mediated by H_1_ and H_3_Rs.

#### Focal seizures

##### Animal data

In a comprehensive study on amygdala kindled seizures in rats, convincing evidence for the suppressive role of central histamine in epilepsy was provided (Kamei, 2001). In the amygdala of kindled rats, a significant decrease of histamine concentrations was disclosed. Exogenous histamine administered to kindled animals elicited the seizure inhibiting effect that was mimicked by H_1_R agonists but not H_2_R agonists. Moreover, when administered repeatedly to rats, L-histidine retarded development of amygdala kindling (Kamei et al., 1998). Histidine and metoprine also inhibited seizures, and both treatments were associated with enhanced histamine levels in cerebral cortex, hippocampus, hypothalamus, and amygdala. H_3_R antagonists evoked an antiepileptic effect, which was prevented by pretreatment with an H_3_R agonist and was sensitive to H_1_R antagonists. As shown by others (Jin et al., 2005) amygdala kindled seizure inhibition could also be achieved by administration of dipeptide carnosine (beta-alanyl-L-histidine). The antiepileptic effect was antagonized by H_1_R blockers of the first generation (pyrilamine, diphenhydramine), indicating histamine participation.

#### Generalized convulsive seizures

Accordingly, manipulations of the endogenous histamine level that resulted in its increase (stimulation of synthesis, inhibition of degradation) was invariably associated with the inhibition of convulsions or increased threshold for seizure induction, the opposite being true for procedures that caused either decrease of brain histamine concentration or blocked histamine signaling via H_1_ or activated H_3_Rs. For instance, metoprine, an inhibitor of histamine catabolizing enzyme, histamine N-methyl transferase, inhibited electroshock seizures (Tuomisto and Tacke, 1986).

##### Animal data

Metoprine inhibited seizures evoked by amygdala kindling in rats (Kamei et al., 1998; Kamei, 2001), as well as reduced audiogenic convulsions in genetically audiogenic seizure sensitive rats (Tuomisto et al., 1987). Likewise, L-histidine inhibited amygdala kindled seizures (Kamei et al., 1998) as well as PTZ-induced seizures in rats (Chen et al., 2002), an effect potentiated by H_3_R antagonist, thioperamide, and antagonized by α-fluoromethylhistidine (inhibitor of histidine decarboxylase) as well as by pyrilamine, H_1_R antagonist (Chen et al., 2002). In audiogenic epilepsy prone Krushinski–Molodkina rats, as opposed to epilepsy resistant Wistar rats, brain histamine concentrations are significantly lower (Onodera et al., 1992). In different animal models of epilepsy, imidazole and non-imidazole H_3_R antagonists facilitating the release of histamine have proven to be beneficial (Yokoyama et al., 1993; Kakinoki et al., 1998; Kamei, 2001). Pitolisant, an H_3_R antagonist, showed excellent antiepileptic activity in animal models of seizure, predictive for generalized, MES test in mice (Sadek et al., 2014).

##### Clinical data

Interestingly, children with febrile seizures showed significantly lower histamine concentrations in cerebrospinal fluid than febrile children without seizures. Based on these findings, the suggestion was made that brain histaminergic system may be involved in inhibiting seizures associated with febrile illnesses in childhood (Kiviranta et al., 1995). The clinical data amply documented proconvulsant effects of H_1_R antagonists administered in clinically relevant doses (Churchill and Gammon, 1949; Yokoyama et al., 1993; Takano et al., 2010; Miyata et al., 2011; Zolaly, 2012). Therefore, centrally acting H_1_R antagonists which may increase seizure susceptibility in patients with febrile seizures are neither recommended to these patients nor to PWE. Also, they should be avoided in young infants, more sensitive to the drugs that could potentially disturb the anticonvulsive central histaminergic system. The potential antiseizure activity of pitolisant, a non-imidazole H_3_R inverse agonist, was examined in 14 photosensitive adults using the photosensitivity standard model and employing 20/40/60 mg dose. Significant suppression of generalized epileptiform discharges was observed in the majority of the PWE (Kasteleijn-Nolst Trenite et al., 2013; Bialer et al., 2015). Unfortunately, a recent multicenter, national, pragmatic, noncomparative, open-label, exploratory phase II trial reported there was no clinical effects of pitolisant in human epilepsy, in spite of the existing promising animal data (Collart Dutilleul et al., 2016).

#### Generalized non-convulsive seizures and epilepsy syndromes

##### Animal data

WAG/Rij strain showed an increase in the density of H_1_R binding in the frontal motor cortex and interposed nucleus of cerebellum and a decrease in the substantia nigra compacta compared to the non-epileptic control group (Midzyanovskaya et al., 2016). Taking into account the bidirectional effect of the H_1_R antagonist pyrilamine on SWDs in WAG/Rij rats (Midzyanovskaya et al., 2005), it may be speculated that histamine modulates different areas involved in opposite absence seizure modulation.

## Monoamines in epilepsy: genetic evidence

### Genetic animal studies

#### Genetics of serotonergic system

As shown in Table 1, various KO animal studies have investigated the contribution of the serotonergic system in the neurobiology of epilepsy (Theodore, 2003; Bagdy et al., 2007).

**Table 1:** **Animal studies investigating the involvement of monoamine systems in epilepsy**.

**Animal model**	**Findings**	**References**
**SEROTONERGIC SYSTEM**		
Genetically epilepsy-prone rats	Deficits in serotonergic and noradrenergic systems	Dailey et al., 1989
	Lower brain 5-HT concentration, synaptosomal 5-HT uptake, and tryptophan hydroxylase activity in regions of forebrain and brainstem	Statnick et al., 1996
	Reduced hippocampal 5-HT receptor density	Dailey et al., 1992; Statnick et al., 1996; Salgado-Commissariat and Alkadhi, 1997
	Antiepileptic drugs such as carbamazepine and valproate increase 5-HT concentrations	Yan et al., 1992; Dailey et al., 1997
Genetic mutant mice lacking 5-HT1A receptors	Increased seizure susceptibility	Sarnyai et al., 2000
Genetic mutant mice lacking 5-HT2C receptors	Increased seizure susceptibility	Tecott et al., 1995; Brennan et al., 1997
C57BL/6J (6J) and C57BL/6ByJ (6ByJ) mice	5-HT2 receptors mediate genetic sensitivity to cocaine-induced convulsions	O'dell et al., 2000
**DOPAMINERGIC AND NORADRENERGIC SYSTEMS**		
Genetic Absence Epilepsy Rats from Strasbourg	Reduced D2 receptor binding sites in the caudate–putamen and CA3 hippocampal region	Jones et al., 2010
Wistar Albino Glaxo rats from Rijswijk	Reduced D2 receptor binding sites in the caudate–putamen and CA3 hippocampal region	Birioukova et al., 2005
D2 receptor knockout (D2R^−/−^) mice	Increased susceptibility to seizures induced by kainic acid	Bozzi et al., 2000; Tripathi et al., 2010
	Increased susceptibility to seizures induced by pilocarpine	Bozzi and Borrelli, 2002, 2006
	CA3 hippocampal apoptotic cell death	Bozzi et al., 2000; Bozzi and Borrelli, 2006; Tripathi et al., 2010
Congenic D4 “knockout” mice	D4 receptors in the interaction with D1 receptors positively regulate D1 receptor-mediated seizures	O'Sullivan et al., 2006
D4 receptor knockout (D4R^−/−^) mice	Spontaneous synaptic activity and epileptic discharges induced by 4-aminopyridine or bicuculline increased in cortical slices	Rubinstein et al., 2001
D1 and D5 receptor knockout mice	D1 and D5 receptor-dependent induction of seizures	O'Sullivan et al., 2008
DBH (DBH^−/−^ mice) knock-out mice	Mice susceptible to of pentylenetetrazole-induced seizures, activation of the a1AR is responsible for the anticonvulsant activity of endogenous noradrenaline; noradrenergic agonists have protective effects against seizures	Weinshenker et al., 2001a
**HISTAMINERGIC SYSTEM**		
H1 receptor gene knockout, histidine decarboxylase deficient and mast cell-deficient mice	Faster development of pentylenetetrazole-induced seizures and increased histamine content in diencephalon	Chen et al., 2003
EL mice-genetic model of human temporal lobe epilepsy	Inhibitory actions of the histaminergic neurons on the epileptogenesis; Pretreatment with histidine and metoprine delayed, while H1 blockade speed up the time of seizure onset	Yawata et al., 2004

Some of the first such studies involved GEPRs, which displayed deficits in serotonergic system (Dailey et al., 1989). Specifically, these rats had lower brain 5-HT concentration, synaptosomal 5-HT uptake, and tryptophan hydroxylase activity in regions of forebrain and brainstem (Statnick et al., 1996). Moreover, various studies demonstrated reduced hippocampal 5-HTR density in the GEPRs (Dailey et al., 1992; Statnick et al., 1996; Salgado-Commissariat and Alkadhi, 1997) suggesting the critical importance of serotonergic activity in seizure regulation (Jobe et al., 1999). In line with these findings, results obtained on GEPRs also suggest that antiepileptic drugs such as carbamazepine and valproate increase 5-HT concentrations as a part of their mechanism of action (Yan et al., 1992; Dailey et al., 1997). In addition to rats, genetic mutant mice lacking 5-HT_1A_Rs (Sarnyai et al., 2000) or 5-HT_2C_Rs (Tecott et al., 1995; Brennan et al., 1997) displayed increased seizure susceptibility, suggesting the involvement of these receptors in the regulation of neuronal excitability. In addition, 5-HT_2C_Rs have been suggested to mediate genetic sensitivity to cocaine-induced convulsions (O'dell et al., 2000).

#### Genetics of dopaminergic and noradrenergic systems

Genetically altered rats and mice were used to provide an insight into the role of the DAergic system in the epileptogenesis (Table 1). DA D_2_R binding sites were found to be reduced in the caudate-putamen and CA3 hippocampal region of GAERS (Jones et al., 2010) and WAG/Rij rats (Birioukova et al., 2005). In mice, inactivation of the D_2_R gene and consequently impaired D_2_R-mediated signaling resulted in more severe seizures. Namely, D_2_R KO mice showed an increased susceptibility to seizures induced by kainic acid (Bozzi et al., 2000) and pilocarpine (Bozzi and Borrelli, 2002, 2006). In these mice, CA3 hippocampal apoptotic cell death was observed (Bozzi et al., 2000; Bozzi and Borrelli, 2006; Tripathi et al., 2010), suggesting that D_2_R activation may be neuroprotective. Further studies on DAR KO mice investigated the intracellular pathways activated by different DARs in response to seizures (Bozzi et al., 2000; Rubinstein et al., 2001; Bozzi and Borrelli, 2002; O'Sullivan et al., 2008; Tripathi et al., 2010; Dunleavy et al., 2013). These experiments also established the role of D_1_ and D_5_Rs in the regulation of synaptic activity (O'Sullivan et al., 2008). Moreover, spontaneous synaptic activity and epileptic discharges induced by 4-aminopyridine or bicuculline were increased in cortical slices from D_4_R KO mice (Rubinstein et al., 2001). It has been suggested that D_4_Rs in interaction with D_1_Rs positively regulate D_1_R-mediated seizures (O'Sullivan et al., 2006).

In the evaluation of the contribution of NA to neuronal excitability (Table 1), an animal model of DBH KO mice was used (Weinshenker et al., 2001b). These mice lacking NA were susceptible to seizures, and noradrenergic agonists showed protective effects against seizures. Endogenous NA had anticonvulsant effects and was confirmed to represent a potent endogenous inhibitor of neuronal excitability, and these results suggest that future strategies should be focused on noradrenergic drugs to treat epilepsy (Weinshenker et al., 2001b).

#### Genetics of histaminergic system

As demonstrated in Table 1, histaminergic neurons have also been postulated to have important role in the inhibition of convulsions and seizures (Yawata et al., 2004), since it has been shown that increased H concentrations suppressed seizures and presumably have neuroprotective properties (Bhowmik et al., 2012). In an animal model of epilepsy with PTZ-induced chemical kindling, behavioral, and neurochemical characteristics were examined in various strains of mutant mice (in H_1_R KO mice, histidine decarboxylase deficient, and mast cell-deficient mice) compared to their wild type mice (Lai et al., 2003). Mutant mice displayed faster development of seizures and increased histamine content in diencephalon compared to the corresponding wild type mice, suggesting that histamine has protective anticonvulsive effects on seizures achieved via H_1_Rs (Lai et al., 2003). In another animal model, in EL mouse (genetic model of human temporal lobe epilepsy), inhibitory actions of the histaminergic neurons on the epileptogenesis were reported (Yawata et al., 2004). In these mice, pretreatment with histidine, a precursor of histamine, and with metoprine, an inhibitor of the histamine N-methyltransferase, delayed the time of onset of the seizures, while H_1_R blockade with antagonist speed it up (Yawata et al., 2004).

### Genetic human studies

#### Genetics of serotonergic system

As shown in Table 2, the genetic background regarding serotonergic system in epileptogenesis has been most frequently investigated using the association between SERT (or 5-HTT) gene variants and epilepsy. Genetic mutations in the 5-HTT gene influence 5-HTT expression and change extracellular 5-HT levels, therefore increasing susceptibility to seizures (Ottman and Risch, 2012; Salzmann and Malafosse, 2012). One of the most studied polymorphisms in this gene is a variable number of tandem repeats (5-HTTVNTR) polymorphism, located in the second intron, with a repetition unit containing 17 bp. There are three alleles of 5-HTTVNTR that contain 9, 10, or 12 repetitions. Less efficient transcriptional genotypes (10/10) of the 5-HTTVNTR were found more frequently in PWE with juvenile myoclonic epilepsy (JME) compared to control subjects of Egyptian origin (Esmail et al., 2015). The similar result, with the higher frequency of the 10-repeat allele of the 5-HTTVNTR in PWE with TLE in comparison to controls, was observed in Brazilian subjects (Schenkel et al., 2011). In Han Chinese population, one study reported higher frequency of the 10-repeat allele (Li et al., 2012), whereas the other found higher frequencies of transcriptionally more efficient 12/12 genotype and allele 12 (Che et al., 2010), in the PWE with TLE than in normal controls. On the other hand, Italian PWE with TLE showed lower frequencies of the 10 repeat of the 5-HTTVNTR in comparison to control subjects (Manna et al., 2007), whereas results obtained on Croatian subjects demonstrated lack of association of 5-HTTVNTR polymorphism with TLE (Stefulj et al., 2010). In subjects suffering from mesial TLE with hippocampal sclerosis (MTE-HS), the treatment response to antiepileptic drug was evaluated: 12/12 genotype of 5-HTTVNTR polymorphism was found to be associated with significantly increased risk for a nonresponse to medical treatment compared to carriers of the 10-repeat allele (Kauffman et al., 2009). Moreover, PWE with TLE carrying the combination of transcriptionally more efficient 5-HTTVNTR (12/12) genotype and L/L genotype of another common 5-HTT polymorphism, 5-HT-transporter-linked polymorphic region (5-HTTLPR), displayed poorer treatment response to antiepileptic medication therapy (Hecimovic et al., 2010). 5-HTTLPR is a biallelic polymorphism located in the 5′ regulatory region of 5-HTT gene. 5-HTTLPR short (S) allele has been associated with lower transcriptional efficiency of this gene and lower 5-HT uptake activity, in comparison to the long (L) allele (Lesch et al., 1994; Heils et al., 1996). Although the homozygous S genotype was found to significantly increase risk of developing alcohol withdrawal seizures and delirium (Sander et al., 1997), no significant differences were observed in the frequencies of 5-HTTLPR genotypes and alleles in PWE with TLE and normal controls in Chinese Han (Che et al., 2010) and Croatian (Stefulj et al., 2010) populations, as well as in the PWE suffering from IGE (Sander et al., 2000). In addition, a meta-analysis including 5-HTTVNTR and 5-HTTLPR polymorphisms, suggested that 5-HTT gene may not be the primary determinant of epilepsy susceptibility, but in the interaction with other genes involved in different signaling pathways, it might participate in epileptogenesis (Yang et al., 2013).

**Table 2:** **Human studies investigating the involvement of monoamine systems in epilepsy**.

**Human study**	**Findings**	**References**
**SEROTONERGIC SYSTEM**		
Juvenile myoclonic epilepsy (JME)	Less efficient transcriptional genotypes (10/10) of the 5-HTTVNTR polymorphism were more frequent in patients with JME	Esmail et al., 2015
Temporal lobe epilepsy (TLE)	Higher frequency of the 10-repeat allele of the 5-HTTVNTR polymorphism in patients with TLE in comparison to controls	Schenkel et al., 2011
	Higher frequency of the 10-repeat allele of the 5-HTTVNTR polymorphism in patients with TLE than in normal controls	Li et al., 2012
	Higher frequencies of transcriptionally more efficient 12/12 genotype and allele 12 of the 5-HTTVNTR polymorphism in patients with TLE than in normal controls	Che et al., 2010
	Lower frequencies of the 10 repeat of the 5-HTTVNTR polymorphism in comparison to control subjects	Manna et al., 2007
	Lack of association of 5-HTTVNTR polymorphism with TLE	Stefulj et al., 2010
	TLE patients carrying the combination of transcriptionally more efficient 5-HTTVNTR (12/12) genotype and L/L genotype of 5-HTTLPR polymorphism had poorer treatment response to antiepileptic therapy	Hecimovic et al., 2010
Mesial temporal lobe epilepsy with hippocampal sclerosis (MTE-HS)	12/12 genotype of 5-HTTVNTR polymorphism associated with increased risk for a nonresponse to medical treatment compared to carriers of the 10-repeat allele	Kauffman et al., 2009
Alcohol withdrawal seizures	Homozygous S genotype of 5-HTTLPR polymorphism significantly increase risk to develop alcohol withdrawal seizures	Sander et al., 1997
Temporal lobe epilepsy (TLE)	Lack of association of 5-HTTLPR polymorphism with TLE	Che et al., 2010; Stefulj et al., 2010
Idiopathic generalized epilepsy	Lack of association of 5-HTTLPR polymorphism with IGE	Sander et al., 2000
Idiopathic generalized epilepsy or alcohol withdrawal seizures	Lack of association of Cys23Ser polymorphism of *HTR2C* with IGE or alcohol withdrawal seizures	Samochowiec et al., 1999; Stefulj et al., 2010
Temporal lobe epilepsy (TLE)	Lack of association of C1019G polymorphism of *HTR1A* with TLE	Stefulj et al., 2010
	Higher expression of 5-HT_1A_ receptor mRNA expression in hippocampal tissue of TLE patients homozygous for the C-allele of rs6295 polymorphism in *HTR1A*, as compared to patients with the GG-genotype	Pernhorst et al., 2013
	Marginally increased frequency of 861G allele of the G861C polymorphism in 5-HT1B receptor gene in the patients with TLE	Stefulj et al., 2010
	T variant of 1354CT polymorphism in *HTR2A* may influence an earlier age of onset of TLE	Manna et al., 2012
**DOPAMINERGIC AND NORADRENERGIC SYSTEMS**		
Epilepsy and antiepileptic drug response	Lack of association between *DBH* C-1021T polymorphism and epilepsy, several epilepsy subtypes, or response to antiepileptic drugs	Depondt et al., 2004
Effects of antiepileptic drug	Patients with genetic variants of *DBH* rs1611115, *COMT* rs4680 and dopamine receptor D2 rs1800497 polymorphisms, associated with decreased dopaminergic activity, have higher susceptibility to negative psychotropic effects of levetiracetam	Helmstaedter et al., 2013
Idiopathic generalized epilepsy (Dailey et al.)	Higher frequency of the 9-copy allele of *DAT* polymorphism was observed in IGE and IAE patients compared to the control group	Sander et al., 2000
Idiopathic absence epilepsy (IAE)		
**ENZYMES INVOLVED IN THE MONOAMINE SYNTHESIS AND METABOLISM**		
Neurological syndrome with learning disabilities, epilepsy, and psychiatric symptoms	Mutation-induced deficiency of the 6 pyruvoyl tetrahydropterin synthase, necessary for normal function of tyrosine and tryptophan hydroxylases	Ng et al., 2015
Neurological syndrome with mental retardation and epilepsy	Inherited duplication of Xp11.3, including *MAOA* and *MAOB* genes	Tzschach et al., 2008; Klitten et al., 2011
	Inherited deletion of Xp11.3, including *MAOA* and *MAOB* genes	Whibley et al., 2010
Idiopathic generalized epilepsies (Dailey et al.)	Lack of association between the *MAOA*-uVNTR polymorphism and different IGE subtypes	Haug et al., 2000
Temporal lobe epilepsy (TLE)	Lack of association between the *MAOA*-uVNTR polymorphism and TLE	Stefulj et al., 2010

Studies investigating the role of 5-HTR gene variants in susceptibility to seizure generation demonstrated no association of Cys23Ser polymorphism located in the gene HTR2C with IGE or alcohol withdrawal seizures (Samochowiec et al., 1999; Stefulj et al., 2010). Moreover, genetic variants of the C1019G polymorphism in the HTR1A gene also displayed similar distribution among PWE with TLE and control subjects (Stefulj et al., 2010). However, the 5-HT_1A_R mRNA expression was found to be higher in hippocampal tissue of PWE with TLE homozygous for the C-allele of rs6295 polymorphism, located in the promoter region of the human HTR1A gene, as compared to PWE with the GG-genotype (Pernhorst et al., 2013). On the other hand, the frequency of 861G allele of the G861C polymorphism in the HTR1B gene was found to be marginally increased in the PWE with TLE, implicating this allele in the susceptibility to TLE (Stefulj et al., 2010). The 861G allele has been linked with fewer 5-HT_1B_Rs in the human brain, in comparison to 861C allele (Huang et al., 1999). In addition, Manna et al. (2012) demonstrated that the T variant of 1354CT polymorphism in HTR2A gene may be implicated in an earlier age of onset of TLE (Manna et al., 2012).

#### Genetics of dopaminergic and noradrenergic systems

DBH, another enzyme involved in conversion of DA to NA, is important for the maintenance of central DA and NA concentrations (Table 2). It is presumed that endogenous NA has an antiepileptic effect, especially in limbic regions, and regulates seizure threshold (Giorgi et al., 2004). Plasma DBH was shown to decrease during epileptic seizures (Miras-Portuga et al., 1975). The functional polymorphism in the *DBH* gene, the DBH (rs1611115 or C-970T or DBH C-1021T) polymorphism affects DBH activity and is responsible for almost 50% of the plasma DBH variations. However, there were no significant differences in the frequency of the TT, TC, and CC genotypes in the DBH C-1021T between large numbers of PWE and control subjects (Depondt et al., 2004). Depondt and colleagues detected no significant association between DBH C-1021T polymorphism and epilepsy, several epilepsy subtypes, or response to antiepileptic drugs, implying that this polymorphism does not contribute to epilepsy (Depondt et al., 2004). On the other hand, epileptic PWE carrying genetic variants of rs1611115 polymorphism in *DBH* gene, rs4680 polymorphism located in the gene coding for catechol-O-methyltransferase (COMT) and rs1800497 polymorphism in DA D_2_R gene, all genetic variants associated with decreased DAergic activity, showed a higher susceptibility to negative psychotropic effects of the antiepileptic drug levetiracetam (Helmstaedter et al., 2013). These findings suggested that decreased DAergic transmission in PWE may worsen the outcome and adverse effects of treatment with specific AEDs.

Genetic studies investigating other components of DAergic system in epilepsy, include polymorphisms in the human DAT gene, which may explain inter-individual differences in the density or affinity of DAT (Table 2). The study of Sander et al. (2000) reported the association of the 40 bp repeats polymorphism in the 3′ untranslated region of the *DAT* gene with IGE, and especially with idiopathic absence epilepsy (IAE) (Sander et al., 2000). Significantly higher frequency of the 9-copy allele of this polymorphism was observed in IGE and IAE PWE compared to the control group (Sander et al., 2000). In addition, various studies demonstrated that the A9 allele (9-copy repeat) of the *DAT* gene contributed to the risk of alcohol-withdrawal seizures and delirium (Sander et al., 1997; Gorwood et al., 2003), suggesting that variations of the *DAT* gene may modulate neuronal excitability and contribute to epileptogenesis.

#### Genetics of enzymes involved in the monoamine synthesis and metabolism

Some insights about the involvement of the human monoaminergic genes in epilepsy have come from a neurological syndrome, which includes learning disabilities, epilepsy, and psychiatric symptoms (Table 2). This syndrome is probably caused by the mutation-induced deficiency of the 6 pyruvoyl tetrahydropterin synthase, necessary for normal function of tyrosine and tryptophan hydroxylases, enzymes enrolled in the synthesis of monoamines (Ng et al., 2015). Moreover, PWE with mental retardation carry an inherited duplication (Tzschach et al., 2008; Klitten et al., 2011), or deletion (Whibley et al., 2010) of Xp11.3, including genes coding for monoamine oxidase A and B (MAO-A and MAO-B), suggested that these genes are important for normal development of the CNS. This is not surprising, as MAO-A and MAO-B play a role in the degradation of monoamine neurotransmitters such as DA, NA, and 5-HT. In PWE with idiopathic generalized epilepsies, like childhood absence epilepsy, JAE, and juvenile myoclonic epilepsy (Blümcke et al.), the functional polymorphism located in the promoter of *MAOA* gene (MAOA-uVNTR) was evaluated to test whether allelic variation has a role in the etiology of IGE (Haug et al., 2000). Although, it was expected that the higher activity promoter alleles (3a and 4 copy alleles) would be associated with susceptibility to epilepsy, the frequencies of the high and low (3 copy allele) activity allele groups were similar between PWE and controls, and these results did not confirm any association between the MAOA-uVNTR polymorphism and different IGE subtypes (Haug et al., 2000). In line with these results, genetic variants of MAOA-uVNTR polymorphism were also similarly distributed among PWE with TLE and control subjects (Stefulj et al., 2010).

Despite the large body of evidence reviewed here, relatively scanty evidence from both animal and human studies is available to support the direct association between epilepsy and variation of genes involved in different aspects of monoaminergic neurotransmission, including synthesis, metabolism, transport, reuptake, or packaging (Ng et al., 2015).

## Monoaminergic strategies to treat epilepsy

As of yet, there are still no fully effective drugs for treating epilepsy. Despite the emergence of new agents, a consistent proportion of PWE remain resistant to drug treatments. It appears clear that Paul Ehrlich's “magic bullets” do not work in complex CNS pathologies such as epilepsy for which “magic shotguns” are instead needed (Roth et al., 2004). Indeed, single-target AED may not always induce the desired effect even if they successfully inhibit or activate a specific target known to be altered in epilepsy (Csermely et al., 2005). One of the expansions is that effectiveness can be affected in compensatory ways. There is a need to develop multi-target anticonvulsants with the ability to prevent or delay the onset of epilepsy and/or the potential for disease modification.

Unfortunately, in contrast to other CNS disorders (i.e., Alzheimer Disorder, AD), a multi-target ligand approach, acting simultaneously on different receptors or enzymatic systems implicated in epilepsy has not yet attracted the attention of medicinal chemists. Specifically, targeting multiple monoamine systems via multi-target-directed ligands (MTDL) may represent a successful approach, since the experimental and clinical evidence reviewed here has demonstrated the pivotal role of different monoaminergic proteins/enzymes in epilepsy. The rational discovery of multi-target drugs this may represent is an emerging area in epilepsy. These drugs may be also useful for the frequent comorbid psychiatric disorders seen in PWE (Bialer and White, 2010; Cardamone et al., 2013).

Some of standard or herbal monoaminergic medicines already show a profile of multi-target drugs acting via modulation of multiple proteins/systems rather than single targets (Di Matteo et al., 2000; Quesseveur et al., 2013), a phenomenon known as polypharmacology (Hopkins, 2008).

Lu et al. (2012) compared the drug targets and the market sales of the new molecular entities approved by the Food and Drug Administration (FDA) using network analysis tools. There are several monoaminergic targets, such as DARs, 5-HTRs, ARs, MAO-B, etc., that are common to the CNS complex diseases, confirming that these targets play crucial roles in the development of complex diseases and in drug discovery (Lu et al., 2012).

### MAO enzymes as targets to treat epilepsy

MAO (EC 1.4.3.4, amine-oxygen oxidoreductase) exists as two isozymes: MAO-A and MAO-B, both showing different substrate specificities, sensitivity to inhibitors, and amino acid sequences. MAO catalyzes the oxidative deamination of a variety of biogenic and xenobiotic amines, with the concomitant production of hydrogen peroxide (Youdim et al., 1988). MAO-A preferentially oxidizes NA and 5-HT and is selectively inhibited by clorgyline, while MAO-B preferentially deaminates β-phenylethylamine and is irreversibly inhibited by *l*-deprenyl (Ramsay, 2013). MAO activity has been shown to be linked to epilepsy since the 1960s (Plotnikoff et al., 1963; Kohli et al., 1967). For instance, MAO-B activity is elevated in hypometabolic regions of PWE with TLE due to activated astrocytes and gliosis (Kumlien et al., 1992), the most common histopathological abnormality seen in this focal epilepsy (Blümcke et al., 2013). PET studies with ^11^C- deprenyl (Kumlien et al., 2001) or autoradiographic studies in human brain slices with ^3^H-deprenyl (Kumlien et al., 1992) have therefore been used for identification of epileptogenic regions in patients with focal epilepsy for surgical resection.

Although, several studies showed that the old MAO inhibitors exhibit anticonvulsant activity (Plotnikoff et al., 1963; Kohen et al., 1996), they have not been used clinically due to their adverse effects. In addition, the magnitude of the anticonvulsant response in animals models vary between MAO inhibitors (MAO-I), while the role of MAO subtypes underscoring the anticonvulsant action of MAO-I is not well understood. More specifically, both selective MAO-A and MAO-B inhibitors (MAO-AIs and MAO-BIs) exert anticonvulsant activity in different preclinical models of seizure (Sparks and Buckholtz, 1985; Mukhopadhyay et al., 1987; Löscher and Lehmann, 1996, 1998; Loscher et al., 1999).

However, the anticonvulsant and the antiepileptogenic effects of *l*-deprenyl, the most extensively studied drug in this respect, seem to be mediated by MAO-A inhibition instead of the irreversible MAO-B inhibition (Loscher et al., 1999). On the one hand, this interpretation agrees with the lack of anticonvulsant efficacy of the selective MAO-BI LU 53439, but potent anticonvulsant activity of the selective MAO-AI esuprone, in the kindling model of epilepsy (Loscher et al., 1999). These results point strongly to MAO-A but not MAO-B inhibition as an effective means of inducing anticonvulsant effects. On the other hand, other MAO-BIs including safinamide or zonisamide have been shown to be efficacious in some seizure models (Bialer, 2012; Park et al., 2015) and zonisamide was approved for epilepsy recently (Bialer, 2012). Zonisamide has been shown to physically interact with human MAO-B, but not MAO-A enzyme (Binda et al., 2011) highlighting that MAO-B inhibition can be worth targeting to achieve an anticonvulsant effect. The fact that MAO-A could indirectly mediate the anticonvulsant properties of MAO-BIs could be related to the complex relationships between MAO-A and MAO-B. Indeed, despite the existence of preferring substrates for each enzyme, the selective blockade of one enzyme has been shown to alter the activity of the other one, particularly after chronic administration (Youdim et al., 2006; Finberg, 2014). In spite of the complex relationships between MAO-A and MAO-B, these studies stress that MAO inhibition may be an interesting strategy for developing novel anticonvulsant agents in considering also the better tolerability of the newer compounds compared to the early MAO-Is (Löscher and Lehmann, 1996, 1998; Loscher et al., 1999; Youdim et al., 2006; Bialer, 2012).

Aside from monoaminergic mechanisms, *l*-deprenyl has been shown to affect the polyamine binding site of the NMDA subtype of GLURs, to stimulate neurotrophic factors, and to modulate gene expression and protein synthesis, which again is unrelated to MAO-B inhibition (Magyar, 2011). Moreover, MAO-BIs seem to be effective by acting on different pathways, along with their enhancing effect on monoaminergic transmission. For instance they possess neuroprotective properties (Aluf et al., 2013) by blocking oxidative stress and ROS formation (Riederer et al., 2004). Accordingly, compelling evidence supports the idea that rasagiline-induced neuroprotection is not related to the inhibition of MAO enzymatic activity. This action has been ascribed to the presence of the reactive propargylamino moiety which might interfere with many other cellular processes such as different key steps of the apoptotic cascade (Al-Nuaimi et al., 2012). In view of these various effects, it is impossible to foresee which effect(s) are most likely to explain the anticonvulsant and antiepileptogenic activity of *l*-deprenyl. Indeed, MAO-BIs show multimodal effects and a MTDL profile (Pisani et al., 2011; Bolea et al., 2013).

To date, the only MTDL strategy targeting monoaminergic systems has focused on MAO inhibition among other targets with cholinesterase (ChE) as a new strategy for AD (Bolea et al., 2013) even if it has not yet led to novel clinical therapeutics (Pisani et al., 2011) with the exception of rasagiline (Youdim, 2003). Interestingly, some drug candidates have emerged from MTDL design showing promising multi-target properties and have been submitted to extensive bio-pharmacological profiling (Cavalli et al., 2008). Members of the Cost ACTION CM1103 (http://www.cost.eu/COST_Actions/cmst/CM1103) have designed, synthetized and evaluated new different MTDL compounds acting on ChE and MAO-I that may elicit better outcomes in the complex nature of AD than the current selective drugs (Benek et al., 2015; Ismaili et al., 2016; Unzeta et al., 2016). Moreover, MAO-Is with additional ion-chelating and/or antioxidant activities, compounds with dual MAO-I and adenosine A2aR antagonist activity have been characterized (Pisani et al., 2011; Guzior et al., 2015). Among these MTDLs based on MAO inhibition, several compounds seem to be promising drug candidates, while others may serve as a valuable inspiration in the search for new effective therapies for epilepsy. Crucial experimental evidence supporting these assumptions is now warranted.

### Monoamine transporters as targets to treat epilepsy

The other important class of monoaminergic drugs that may be useful for treating epilepsy is the monoamine transporters (MATs). MATs (DAT, SERT, and NA transporter or NAT) are transmembrane proteins located in plasma membranes of monoaminergic neurons, while SERT is also expressed in platelets (Amara and Kuhar, 1993). Due to amino acid sequence and proposed structural similarity among the three plasma membrane transporters, many MAT inhibitors have affinity for all three transporters.

Antidepressants are commonly prescribed to PWE to treat comorbid depression and/or anxiety. These include selective serotonin reuptake inhibitors (SSRIs), serotonin-noradrenaline reuptake inhibitors (SNRIs), and related medications (Cardamone et al., 2013). Strikingly, several preclinical and human studies have shown that antidepressants have an anticonvulsant effect and in PWE can improve seizure outcomes, with some patients experiencing dramatic and complete seizure freedom during antidepressant treatment (see Cardamone et al., 2013 for recent review of the literature). Mounting experimental and clinical evidence indicates that antidepressants are anticonvulsants, not proconvulsants as was earlier believed (Jobe and Browning, 2005). Indeed, the proconvulsant effects of antidepressants are mainly reported in cases of overdose, or when therapeutic relevant doses are excessive for slow metabolizers (Preskorn and Fast, 1992). Nevertheless, this erroneous convulsant liability of antidepressants has hindered their use in epilepsy.

SSRIs and SNRIs selectively inhibit monoamine reuptake at the neuronal presynaptic membrane by blocking the 5-HT and 5-HT/NA reuptake transporters, respectively, increasing 5-HT and/or NA levels in the synapse and in the peri-extrasynaptic space. Serotonin and NA may therefore modulate neuronal (i.e., excitability and release of other neurotransmitters) and neuroglial activity. Different lines of enquiries have indicated that SSRIs and SNRIs possess a wealth of potentially therapeutic targets apart from simply increasing monoamine in the CNS, including anti-inflammatory, antioxidant, neuroprotective, and immunomodulatory effects, increase in the brain-derived neurotrophic factor (BDNF), and modulation in the mTOR pathway (Dale et al., 2015).

This list is by no means exhaustive, and other processes, including genetic/genomic and epigenetic mechanisms may be equally important. SSRIs and SNRIs may impact on the different neurobiological alterations occurring in epileptogenesis, and may potentially influence disease course. Alper et al. (2007) reviewed the effects of some SSRIs and SNRIs on seizure incidence in a large cohort of non-epileptic patients in phase II and III of FDA clinical trials of depression treatment between 1985 and 2004. Among the outputs of this study, it appeared that the incidence of seizures occurring in depressed patients treated with antidepressants was significantly lower, compared to those treated with a placebo (Alper et al., 2007). Unfortunately, there have been no double-blind, randomized controlled studies yet; most of the studies have been small and on highly selected patient populations recruited from epilepsy clinics or following epilepsy surgery, and with few longitudinal, follow-up studies.

It is possible that SSRIs and SNRIs, by targeting mechanisms that are both involved in seizure generation and psychiatric comorbidities, may induce both seizure suppression and antidepressant effects. This multi-target profile possessed by SSRIs and SNRIs provides them with the potential to meet some of the criteria for MTDLs. The introduction of drugs such as duloxetine for the treatment of major depression (Carter and McCormack, 2009) indicates the clinical feasibility of designing multifunctional ligands to treat CNS disorders with complex disease pathways, such as epilepsy. Another example is a class of compounds known as triple reuptake inhibitors (i.e., amitifadine) that simultaneously block the synaptic reuptake of 5-HT, NA, and DA (SNDRIs; Skolnick et al., 2006; Weng et al., 2015). Again, as for the MAO-based MTDLs experimental evaluation in animal models of epilepsy and in PWE is needed.

### Monoamine receptors as targets to treat epilepsy

A more successful approach with fewer side effects would be to selectively target some monoaminergic receptors instead of increasing monoamine concentrations with MAO and/or MAT inhibitors treatment. Based on the evidence reviewed here, it might be inferred that the development of MTDLs with optimal polypharmacological profile would exhibit, for example, agonistic activity at 5-HT_2C_Rs/D_2_Rs/α_2_-ARs and antagonistic activity at H_3_Rs but also antagonistic effects at 5-HT_2C_Rs/MT_1/2_Rs (agomelatine). Polypharmacological approaches are therefore likely to be extensively applied for rational design of ligands with optimal multitarget profile and for the discovery of multipotent drug candidates with improved efficacy and safety in therapy of complex brain diseases (Nikolic et al., 2016).

## New research trends in monoaminergic strategies to treat epilepsy

Epilepsy is no longer believed to be strictly a disturbance in the functioning of neurons and specifically of their contact points, the synapses. Instead, it is now seen as an imbalance of the physiological extracellular milieu, due to a plethora of different mechanisms. Therefore, the final imbalance between excitation and inhibition in synaptic transmission that underlies hyperexcitability of the epileptic brain (see van Gelder and Sherwin, 2003 for a review) is far from being a mere direct alteration of the excitatory glutamatergic and the inhibitory GABAergic neurotransmission. Indeed, the pathophysiology underlying ictogenesis and the development of epilepsy is very complex, and clearly does not involve only neuronal cells. Much recent evidence points to a significant contribution made by glial cells to the pathophysiology of epilepsy (Devinsky et al., 2013). This follows the new concept that glial cells interact closely with neurons and play an active role in brain functions. Astrocytes, the major type of glia, make direct contact with neurons via a structure that has been defined as the *tripartite synapse*, in which the astrocytic process is associated with the pre- and post-synapse areas of neurons (Araque et al., 1999). Many normal astrocytic functions are depressed in epilepsy, including K^+^ homeostasis and accompanying changes in aquaporin, gap-junction expression and function, local blood flow and the blood-brain barrier (BBB), uptake and metabolism of GLU and glucose in astrocytes, and neurotransmitter supply, particularly in inhibitory neurons (see Devinsky et al., 2013; Coulter and Steinhauser, 2015 for extensive reviews). Moreover, glial GABA transporter-1 (GAT-1) activity in thalamic astrocytes is impaired in absence epilepsy causing the enhanced GABA levels typical of this generalized non convulsive epilepsy (Richards et al., 1995) and leading to an aberrant GABA_A_ receptor-mediated tonic inhibition (Cope et al., 2009; Pirttimaki et al., 2013).

Emerging evidence suggests that also microglia cells, the CNS resident macrophage cells, play important physiological roles at synapses to such as extent that the concept of a quad-partite synapse has been recently suggested (Schafer et al., 2013).

It is instead well-known that inflammation is due to microglia activation and linked to different CNS diseases. For instance, a large body of evidence indicates that inflammatory changes sustained by uncontrolled glial-mediated immunity contribute to epileptogenesis (Devinsky et al., 2013). Moreover, activated microglia promote astrocytic activation and *vice versa* (Liu et al., 2011), thus sustaining a vicious circle. All these biochemical changes, often linked with gliosis, have significant functional consequences, contributing to epileptogenesis.

Considering the fact that monoamines modulate human behaviors and CNS functions, the antiepileptic, and anticonvulsant action of monoamine ligands may be due to their effect on different targets.

It is well-known that monoamines classically modulate cell excitability by controlling release of GLU, GABA and, other neurotransmitters and ion channels as we have reviewed (for detailed reviews see also Ciranna, 2006; Fink and Goethert, 2007). However, it is a relative new evidence that astrocytes contribute in the cellular action of antidepressants (Schipke et al., 2011; Bernstein et al., 2015; Hertz et al., 2015). Moreover, as further proof of a glial dysfunction in depression, decreased density and number of glia cells has been observed in cortical regions, including the prefrontal and cingulate areas in humans (Rajkowska and Stockmeier, 2013), In addition, selective destruction of frontocortical astrocytes (Banasr and Duman, 2008), NG2-expressing glia (NG2 glia) in the prefrontal cortex (Birey et al., 2015) or pharmacological and genetic inhibition of the activity of the glial glutamate transporter GLT-1 in subcortical areas i.e., the lateral habenula (Cui et al., 2014), were capable of triggering a depressive-like phenotype in rodents.

Astrocytes express virtually all of the receptor systems and ion channels found in neurons (Verkhratsky and Kettenmann, 1996) including transporters critical for synaptic uptake of glutamate (Tanaka et al., 1997) and GABA (De Biasi et al., 1998). Monoamine receptors, (Azmitia et al., 1996), such as 5-HT_2A/2B/2C_ receptors are expressed on astrocytes (Hirst et al., 1998; Sanden et al., 2000; Hwang et al., 2008), but also 5-HT_4_, 5-HT_5_, and 5-HT_7_ receptors (Quesseveur et al., 2013), α-ARs (Bekar et al., 2008) and β_2_-ARs (Mantyh et al., 1995), and all the DA receptors (Miyazaki et al., 2004) are detected. The SERT, NAT, DAT, and the catabolic isoenzymes responsible for the degradation of monoamines (i.e., MAO-A and MAO-B; COMT) were clearly identified in this cell type (see Quesseveur et al., 2013 for a recent review and references within). These observations emphasize the fact that astrocytes can regulate the extracellular monoamine levels by modulating the expression and function of MAO-A, MAO-B, and COMT and at the same time are regulated by feedback by monoamines via the glial monoamine receptors.

SSRIs citalopram and fluoxetine can excite astrocytes directly by inducing astrocytic calcium transients that, differently from the glutamate-induced calcium responses in astrocytes, occur time-delayed, asynchronously and sometimes in an oscillatory manner (Schipke et al., 2011).

Monoamines can change expression of various molecules (Shishkina et al., 2012) involved in epileptogenesis acting on microglial cells. The link between monoamines and microglia is bidirectional. Indeed, microglial pro-inflammatory cytokines, levels of which increase during epileptogenesis, can decrease 5-HT, DA, and NA availability by acting on their presynaptic reuptake transporters through activation of mitogen-activated protein kinase pathways (Zhu et al., 2010) and by reducing monoamine synthesis through decreasing enzymatic co-factors such as tetrahydrobiopterin (Neurauter et al., 2008). Moreover, many cytokines activate the enzyme indoleamine 2,3-dioxygenase which converts tryptophan into kynurenine (Maes et al., 2011).

**Figure 1:**
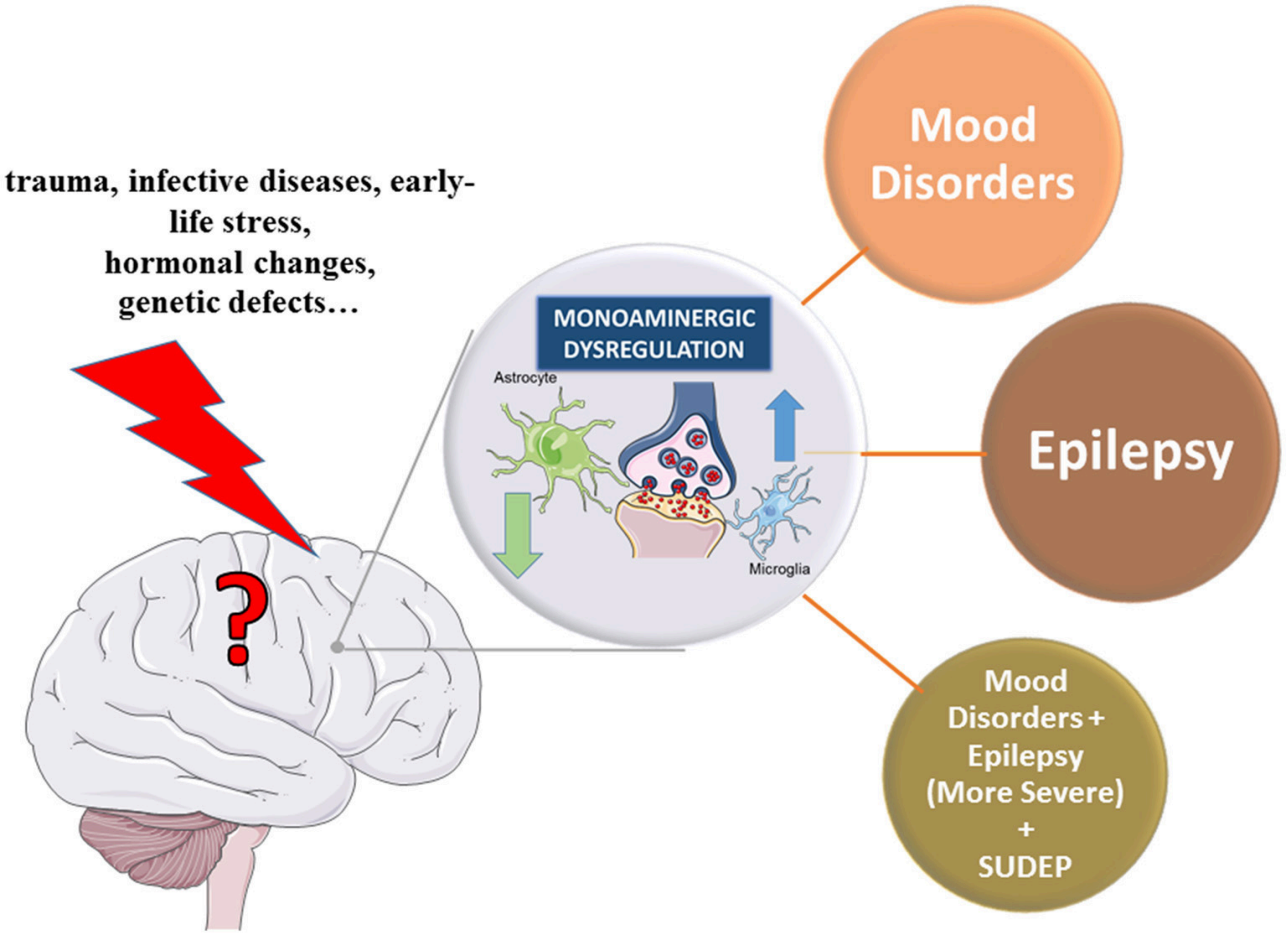
**Hypothetical monoaminergic quad-partite synapse dysfunction as a common pathological mechanism of mood disorders and epilepsy**. The processing of information in synapses is not only defined by neurons, but also by glia cells, namely by astrocytes, which enwrap synapses, and microglia, which dynamically interact with synapses in an activity-dependent manner. This new evidence has brought the development of the quad-partite synapse model, as a further evolution of the tripartite synapse, made up of four elements i.e., presynaptic and postsynaptic neuronal terminals, astrocyte and microglia cells (Schafer et al., 2013). Numerous lines of evidence support the contention that a modification of the quad-partite synapse astrocytes and microglia in different brain regions is associated with depression, and epilepsy (Crunelli and Carmignoto, 2013; Quesseveur et al., 2013). We propose that there may be shared underlying pathology that predisposes patients to depression, epilepsy or both seizures and depression (the latter “seizure/depression phenotype”). For example, traumas, infective disease, early life stress, hormonal changes, genetic and developmental defects, just to cite a few, might induce a dysfunction of the monoaminergic quad-partite synapse and different pathogenic scenarios might cause depression, epilepsy or both conditions. The underlying pathology in the monoaminergic systems of patients with epilepsy (PWE) lowers the threshold for seizures, while also increasing the risk of depression. Moreover, PWE suffering of mood disorders have a higher risk to develop severe and drug-resistant epilepsy (Kanner et al., 2003) and sudden unexpected death in epilepsy (SUDEP; Richerson and Buchanan, 2011). Arrows indicate hypo- or hyperfunction of the glial cells.

From the analysis of the experimental and clinical evidence reviewed here it is possible to hypothesize that a dysfunction of the monoaminergic quad-partite synapse due to an insult (i.e., neurotrauma, infectious injury, genetic disorders…) may be a common pathophysiological mechanism of epilepsy and mood disorders. Depending on the alterations of the quad-partite synapse functions we might have particular mood disorders, epilepsy, or neuropsychiatric disorders with epilepsy (or *vice versa*?). In the latter scenario, depression may be a biologic marker for more severe epilepsy since it is a predictor of a worse seizure outcome in PWE with TLE (Kanner et al., 2003; Figure 1). Some of the antiepileptic/antiepileptogenic effects exerted by monoamines might be due to their ability to act on glia targets. For instance, we have suggested that Ro 60-0175 may normalize the aberrant enhanced GABA_A_ tonic current of the thalamic neurons of GAERS (Cavaccini et al., 2012) by activating astrocytic 5-HT_2C_Rsthat increase activity of glial GAT-1 transporter leading to a reduction of extrasynaptic GABA levels. This hypothesis reinforce the idea of a glial dysfunction in epilepsy and glial cells as new therapeutic targets for this disorder (Crunelli and Carmignoto, 2013; Crunelli et al., 2015). Nevertheless, the role of 5-HT in the function of glial GABA transporters and in general in the modulation of GABA homeostasis, although earlier suggested (Voutsinos et al., 1998), remains to be fully investigated in epilepsy. Interestingly, other early findings showed that 5-HT is capable of modulating the activity of glial Na^+^/K^+^-ATPase but not in the kindled glial fraction (Hernandez and Condes-Lara, 1989). The failure of this 5-HT control of reactive astrocytes due to repeated seizures may contribute to seizure-like activity and epileptogenesis.

## Conclusion

In this review, recent evidence from both animal and human studies supporting the role of monoamines in epilepsy was described. The possible therapeutic application of these findings has been long disregarded, mainly due the severe side effects of some monoaminergic drugs or due to interpretative bias on research evidence.

As further reasons of this stall are that the both the pathophysiological mechanisms underlying epileptogenesis and the genetics of epilepsy are still not clear. Therefore, further research on different aspects of monoaminergic neurotransmission using human genetic biomarkers in combination with novel animal genetic models, might elucidate the complex role of monoamines in the pathophysiology of epilepsy and might accelerate development of novel therapeutic strategies targeting various components of monoaminergic systems. This is desperately needed, firstly because the number of anti-seizure drugs in clinical development has been decreasing over the years (Bialer et al., 2013) and secondly for the increasing number of PWE with refractory epilepsy. Therefore, the beneficial effects of ligands at D_2_R, 5-HT_2C_R, MTRs, α-ARs, H_3_Rs, MAO-Is, and MTAs inhibitors observed in both animal and human epilepsy would deserve more attention both from preclinical and clinical researchers and above all medicinal chemists.

In our opinion, adequate control of epileptogenesis and convulsions and comorbid psychiatric disorders will likely benefit from MTDLs that target different synergistic monoaminergic pathways and different elements of the quad-partite synapse. Nevertheless, their synthesis and experimental validation needs significant efforts and time. There is a scientific and economic incentive for their synthesis as anti-epileptic and/or anti-epileptogenic drug targets. New monoaminergic drugs with fewer side effects and a multi-target profile are warranted.

## Author contributions

All authors contributed to the conception and interpretation of the work and to its critical revision. All authors have approved the final version and may be held accountable for the integrity of this review of current literature.

### Conflict of interest statement

The authors declare that the research was conducted in the absence of any commercial or financial relationships that could be construed as a potential conflict of interest.

## Abbreviations

SERT: 5-HT transporter
5-HTTLPR: 5-HT-transporter-linked polymorphic region
6-OHDA: 6-hydroxydopamine
AC: adenylyl cyclase
AR: adrenoceptor
AT1: angiotensin
AEDs: antiepileptic drugs
DBH: beta-hydroxylase
SNDRIs, block the synaptic reuptake of 5-HT: NA and DA
BDNF: brain-derived neurotrophic factor
beta-alanyl-L-histidine: carnosine
COMT: catechol-O-methyltransferase
CNS: Central Nervous System
CSF: cerebrospinal fluid
ChE: cholinesterase
cAMP: cyclic adenosine monophosphate
DAT: DA transporter
DBA: Dilute Brown Non-Agouti
DA: dopamine
FDA: Food and Drug Administration
GAT-1: GABA transporter-1
GAERS: Genetic Absence Epilepsy Rats from Strasbourg
GEPR: genetic epilepsy-prone rat
GLU: glutamate
HRs: histamine receptors
IAE: idiopathic absence epilepsy
JME: juvenile myoclonic epilepsy
KA: kainic acid
KO: knock out
L-DOPA, L-3: 4-dihydroxyphenylalanine
L: long
MAO-I: MAO inhibitors
MAO-BIs: MAO-B inhibitors
MES: maximal electroshock seizure
mTOR: mechanistic target of rapamycin
MT: melatonin
MTE-HS: mesial TLE with hippocampal sclerosis
mCPP: meta-chlorophenylpiperazine
MAO-A and MAO-B: monoamine oxidase A and B
MATs: monoamine transporters
MTDL: multi-target-directed ligands
DSP4: N-(2-chloroethyl)-N-ethyl-2-bromobenzylamine
NAT: NA transporter
NMDA: N-methyl-d-aspartate
PTZ: pentylenetretrazole
PWE: people with epilepsy
PLC: phospholipase C
PET: positron emission tomography
SSRIs: selective serotonin reuptake inhibitors
5-HT: Serotonin
SNRIs: serotonin-noradrenaline reuptake inhibitors
S: short
SCN1A: sodium voltage gated channel alpha subunit 1
SCN2A: sodium voltage-gated channel alpha subunit 2
SRSs: spontaneous recur rent seizures
SE: status epilepticus
TLE: temporal lobe epilepsy
SUDEP: unexpected death in epilepsy
VNS: vagus nerve stimulation
WAG/Rij: Wistar Albino Glaxo rats from Rijswijk.
